# MafFilter: a highly flexible and extensible multiple genome alignment files processor

**DOI:** 10.1186/1471-2164-15-53

**Published:** 2014-01-22

**Authors:** Julien Y Dutheil, Sylvain Gaillard, Eva H Stukenbrock

**Affiliations:** 1 Max Planck Institute for Terrestrial Microbiology, Department of Organismic Interactions, Marburg, Germany; 2 Université Montpellier 2, CNRS UMR 5554, Institut des Sciences de l’Evolution de Montpellier, Place E. Bataillon, Montpellier 34095, France; 3 INRA, UMR 1345 Institut de Recherche en Horticulture et Semences, SFR 4207 QUASAV, Angers F-49045, France; 4 Agrocampus Ouest, UMR 1345 Institut de Recherche en Horticulture et Semences, SFR 4207 QUASAV, Angers F-49045, France; 5 Université d’Angers, LUNAM Université, UMR 1345 Institut de Recherche en Horticulture et Semences, SFR 4207 QUASAV, Angers F-49045, France; 6 Max Planck Institute for Terrestrial Microbiology, Fungal Biodiversity Group, Marburg, Germany

## Abstract

**Background:**

Sequence alignments are the starting point for most evolutionary and comparative analyses. Full genome sequences can be compared to study patterns of within and between species variation. Genome sequence alignments are complex structures containing information such as coordinates, quality scores and synteny structure, which are stored in Multiple Alignment Format (MAF) files. Processing these alignments therefore involves parsing and manipulating typically large MAF files in an efficient way.

**Results:**

MafFilter is a command-line driven program written in C++ that enables the processing of genome alignments stored in the Multiple Alignment Format in an efficient and extensible manner. It provides an extensive set of tools which can be parametrized and combined by the user via option files. We demonstrate the software’s functionality and performance on several biological examples covering Primate genomics and fungal population genomics. Example analyses involve window-based alignment filtering, feature extractions and various statistics, phylogenetics and population genomics calculations.

**Conclusions:**

MafFilter is a highly efficient and flexible tool to analyse multiple genome alignments. By allowing the user to combine a large set of available methods, as well as designing his/her own, it enables the design of custom data filtering and analysis pipelines for genomic studies. MafFilter is an open source software available at http://bioweb.me/maffilter.

## Background

**Figure 1:**
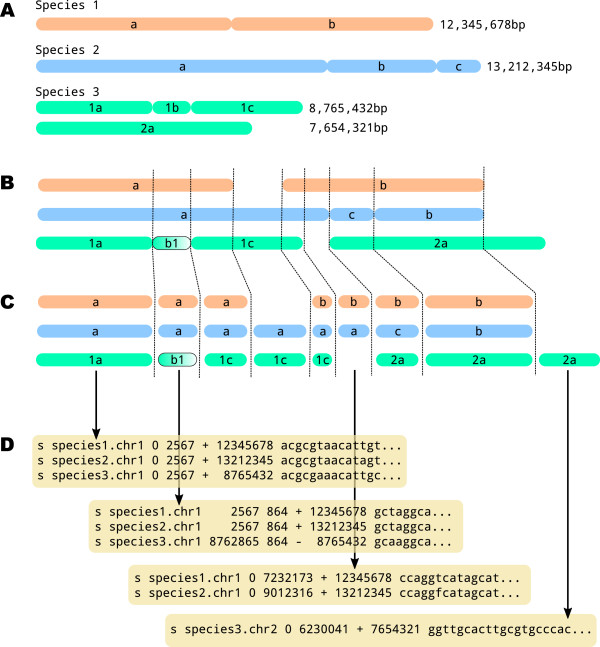
**Genome alignment. A)** Schematic karyotypes of three species and length of their chromosomes. **B)** Chromosome alignment of the three species. Species 2 displays a rearrangement (segments b and c are inverted), and species 3 has an inversion (segment 1b). **C)** Corresponding synteny blocks. **D)** Synteny blocks written in the MAF format.

Evolutionary comparative genomics and population genomics analyses build on alignments of genome sequences that record homologous nucleotide positions between two or more genomes. While gene alignments are described with only three types of character edit (mismatches, insertions and deletions), genome alignments allow for rearrangements, such as inversions, complementations and translocations, leading to breakage of at least one of the input sequence. Regions devoid of such breaks are called synteny blocks, and a genome alignment can be defined as a set of such blocks of homologous positions (Figure 1).

Multiple alignment files are used for storing and sharing genome comparison data. They are typically written in the Multiple Alignment Format (MAF, see Figure 1D), a format in particular popularized by the UCSC genome browser [1]. Programs generating MAF files include BlastZ and MultiZ from the Threaded Blockset Aligner (TBA) package [2] or Last [3]. The multiple alignment serves as an entry to further analyses and several processing steps are required to filter the data, particularly the removal of low-quality regions. In addition, many downstream analysis tools take as input single syntenic blocks only, requiring the global alignment to be exported into multiple alignment files in an external format such as Fasta or Phylip. This conversion often comes at the cost of losing information such as original genome coordinates that may be required in the further analysis pipeline. Solutions to this issue can involve the generation of a database that integrates all analyses results [4]. This is however a tedious process, which conveniently can be avoided.

**Figure 2:**
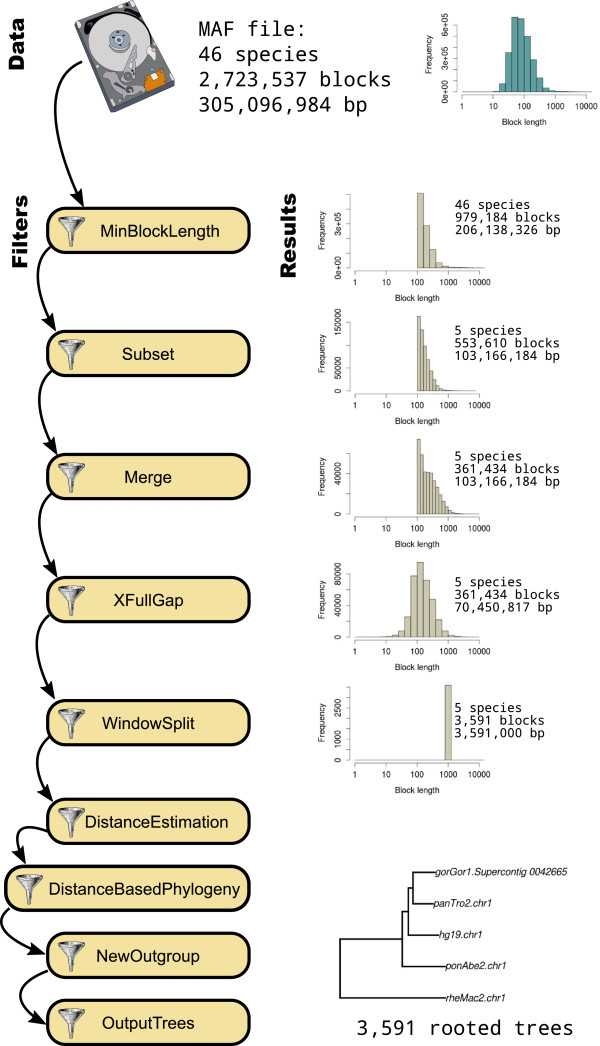
**Example pipeline.** The UCSC 46 vertebrate genome alignment of Human chromosome 1 is processed through several filters in order to reconstruct the genealogy of the Primate sequences in windows of 1kb. The distribution of block sizes at various steps of the filtering is shown under **Results**.

We here introduce a new program which facilitates the processing and analysis of multiple alignment files. The program allows the user to define his/her own analysis pipeline and efficiently processes the input MAF file. Each synteny block is processed separately and passed through a series of *filters* previously defined by the user (see Figure 2). Each *filter* performs a predefined task, such as cleaning the alignment or computing relevant statistics, and passes the synteny block, eventually modified, to the next filter. The output of the program is typically one or several MAF files, and/or several alignment files in external formats like Fasta or Phylip. Furthermore the program allows the output of statistical results in spreadsheet files, or even tree files if phylogenetics analyses are involved. MafFilter deals internally with sequence meta-data such as genome coordinates or quality scores, thereby facilitating the analysis of final results and their integration with external sources of information.

## Implementation

### Program interface

**Figure 3:**
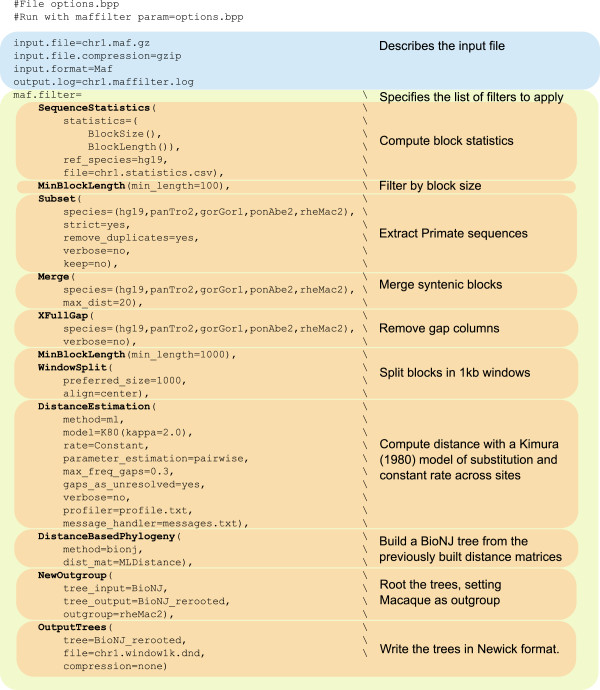
**Example option file.** The MafFilter program is controlled via options with format key=value that can be passed directly as argument of the program or stored into a parameter file. The simple parameter file exemplified here generates the pipeline depicted on Figure 2.

The MafFilter program provides a command line interface to the MAF parser and filters from the Bio++ libraries. It uses the Bio++ Options language, a 'key-value’ syntax introduced for the Bio++ Program Suite which features strong similarities with the R statistical language [5,6]. Briefly, the behaviour of MafFilter is controlled via commands of the type option=value, which can be passed as arguments to the program, or stored in an independent option file. In the simplest case, values are simple numbers or expressions, but can be of more complex structures with arguments. Figure 3 shows the option file generating the pipeline displayed in Figure 2. The first lines describe the input file (path, format and compression). Subsequently is given a list of filters, defining the various steps in the pipeline (see Table 1 for available filters). Each filter accepts several arguments allowing the user to specify parameters and output files. The special filter SequenceStatistics allows the computation of several statistics (Table 2) which are specified by the user. Some of these statistics accept additional control arguments such as a subset of species, or ingroup and outgroup specifications.

**Table 1:** Available filters

**Filter name**	**MafIterator class**	**Characteristics**
**Data extraction**		
Subset	SequenceFilterMafIterator	Extract alignments for a given set of species, filter duplicates.
Merge	BlockMergerMafIterator	Merge consecutive blocks if they are syntenic.
Concatenate	ConcatenateMafIterator	Concatenate consecutive blocks up to a given length, regardless of coordinates.
XFullGap	FullGapFilterMafIterator	Remove gap positions for a given ingroup.
FeatureFilter	FeatureFilterMafIterator	Remove regions from a given feature file.
ExtractFeature	FeatureExtractor	Remove regions outside the ones specified in a given feature file.
SelectChr	ChromosomeMafIterator	Extract alignments from a given chromosome.
**Block filtering**		
MinBlockLength	BlockLengthMafIterator	Remove alignment blocks with too little sites.
MinBlockSize	BlockSizeMafIterator	Remove alignment blocks with too little sequences.
**Sliding window-based alignment processing**		
AlnFilter	AlignmentFilterMafIterator	}Remove ambiguously aligned regions.
AlnFilter2	AlignmentFilterMafIterator2	
EntropyFilter	EntropyFilterMafIterator	Remove highly variable regions.
MaskFilter	MaskFilterMafIterator	Remove masked regions.
QualityFilter	QualityFilterMafIterator	Remove regions with low quality.
**Statistical analysis**		
WindowSplit	WindowSplitMafIterator	Split blocks into windows of given size.
SequenceStatistics	SequenceStatisticsMafIterator	Compute a user-defined selection of statistics, such as character frequencies, alignment size and length, frequency spectrum, counts of fixed vs. polymorphic sites and pairwise divergences. Results are output to a CSV file together with block coordinates for subsequent analysis.
DistanceEstimation	(several classes) ^*a*^	Estimate distance matrix between species.
DistanceBased-Phylogeny	DistanceBasedPhylogenyReconstructionMafIterator	Reconstruct block-wise phylogenies using distance-based methods (W/UPGMA, Neighbor joining, BioNJ)
NewOutgroup	NewOutgroupMafIterator	Reroot a block-wise phylogenetic tree according to a given species.
DropSpecies	DropSpeciesMafIterator	Remove leaves from block-wise trees for agiven species.
**Output**		
Output	OutputMafIterator	Write blocks to a file in MAF format.
OutputAlignments	OutputAlignmentMafIterator	Write blocks into an alignment file, forinstance Fasta or Clustal, using Ensemblsyntax for storing coordinates.
OutputTrees	OutputTreeMafIterator	Write associated trees to a Newick file.
VcfOutput	VcfOutputMafIterator	Call SNPs for each block and write them in a VCF file [17].

^*a*^depending on the method used.

**Table 2:** Available statistics

**Statistic name**	**MafStatistic class**	**Characteristics**
**Block structure**		
BlockLength	BlockLengthMafStatistics	Report the alignment length of each block.
SequenceLength	SequenceLengthMafStatistics	Report the number of nucleotides of a given species in each block.
BlockSize	BlockLengthMafStatistics	Report the number of sequences in each block.
AlnScore	AlignmentScoreMafStatistics	Report the alignment score for each block, if any.
**Block content**		
BlockCounts	CharacterCountsMafStatistics	Compute the frequency of each character in each block.
SiteStatistics	SiteMafStatistics	Compute site-base statistics: number of sites without gap, number of complete sites (no gap, no unresolved character), number of parsimony-informative sites in each block.
**Population genetics**		
PairwiseDivergence	PairwiseDivergenceMafStatistics	Compute the sequence dissimilarity between two individuals.
PolymorphismStatistics	PolymorphismMafStatistics	Compare two groups of sequences, and compute the number of fixed/polymorphic sites between and within each group.
DiversityStatistics	SequenceDiversityMafStatistics	For a given group of sequences, compute the number of seggregating sites and Watterson’s theta.
SiteFrequencySpectrum	SiteFrequencySpectrumMafStatistics	Compute the (unfolded) frequency spectrum for a given group of sequences.
CountClusters	CountClustersMafStatistics	Compute the number of haplotype groups, given a certain mutation threshold, providing a tree has been previously computed.

### Code design

The central data elements in a genome alignment are synteny blocks, *i.e.* contiguous genomic regions sharing common ancestry represented as a sequence alignment. A genome alignment consists of a collection of these blocks together with the corresponding coordinates for each single genome. We developed new data structures for handling such data. Each synteny block is stored as a MafBlock instance which stores the underlying alignment into a SiteContainer, a central class of the Bio++ library for which numerous methods and tools are already available [7]. Individual sequences are stored as MafSequence objects, an extension of the SequenceWithAnnotation class from the *bpp-seq* library allowing the storage and processing of associated quality scores. In addition, MafSequence stores genomic coordinates as chromosome names, strands and start positions.

To process the input genome alignment, MafFilter uses a streaming strategy, as storing all alignment blocks into memory would be highly inefficient, if ever possible, for large data sets. We developed an iterator-based implementation, which loops over all blocks in a file while storing only the necessary information in memory. This is achieved through the new MafIterator classes, which retrieve the next available block when calling the nextBlock method. The use of iterator classes permits to easily implement complex processing procedures as workflows. We name “filter” a special instance of MafIterator which takes as input (typically via the constructor of the class) another instance of MafIterator. Calling the nextBlock method of the filter will automatically call the nextBlock method of the input MafIterator. Looping on the final iterator will thereby automatically loop over all input blocks. As a filter can input another filter it is possible to design a complete processing chain in an easy and highly modulable way.

Table 1 lists all currently available MafIterator classes. One of the classes implementing the MafIterator interface is the MafAlignmentParser itself, which iterates over all blocks in a MAF file. Conversely, the OutputMafIterator class takes as input another MafIterator and writes all available blocks to a file in the MAF format. Finally, the SequenceStatisticsMafIterator applies a series of user-defined statistics on each block, before forwarding it without modification. Usable statistics (see Table 2 for list of currently available statistics) implement the MafStatistics interface. Adding new processing steps to MafFilter is made easy by this object-oriented, iterator-based implementation as the developer only has to provide a new implementation of the MafIterator or MafStatistics interfaces. These new C++ classes can also be used for developing new software independently of MafFilter and we therefore distribute them as part of the Bio++ *bpp-seq-omics* library [6].

### Computer efficiency

As MAF files can be rather large (typically several gigabytes) MafFilter can read and write compressed files, using the *zip*, *gzip* and *bzip2* compression formats. The compression and decompression is achieved with the boost-iostream library. Practically, the use of compressed files has very little impact on the memory usage or computation speed while reducing considerably the amount of disk space. At the time of writing, the amount of publicly available parsers for MAF files is rather limited. The corresponding classes in the Python language have not yet integrated the stable branch of the BioPython libraries. In order to assess the performance of the Bio++ parser, we therefore compare it to the BioPerl library. The resulting perl script (see Additional file 1) parses the compressed MAF file and outputs for each alignment block with more than a thousand sites the number of sequences, the length of the alignment and the coordinates of the sequence of one species if represented in the alignment block. This simple pipeline allows to directly compare the efficiency of the parsers themselves, as the only computations required are file reading, as well as allocation and initialization of the dedicated structures for storing data into memory. The corresponding MafFilter option file is provided in the example directory of the distributed source code. To compare the two approaches, we used the 46 vertebrates alignment of Human chromosome 22 downloaded from UCSC [8] as input data, and ran the analyses on a linux workstation (Intel(R) Xeon(R) CPU E5520 @ 2.27GHz, with 16Gb of RAM running Ubuntu 12.04). The complete parsing takes 30 minutes with the BioPerl script while it completes in only 3 minutes with MafFilter. MafFilter was used to analyse the complete Gorilla genome aligned with other Primates (2Gb alignment) [9], as well as resequencing data of 27 individual genomes from the fungus *Zymoseptoria pseudotritici* (40Mb alignment, E. Stukenbrock, pers. communication).

## Results

### Approximate the ancestral recombination graph with 1kb windows

We downloaded the 46 vertebrate genome alignment from UCSC [8] and built a pipeline in order to infer underlying sequence genealogy along the genome (Figure 2). The tasks performed by MafFilter involved: (1) extracting the Primate sequences (Human, Chimp, Gorilla, Orangutan and Macaque), (2) merging syntenic blocks in Primates, (3) removing gap positions in each block, (4) cutting the resulting alignment into windows of 1kb with no synteny break and (5) computing a distance tree with Kimura distance [10] in each window. The analysis of chromosome 1, the largest alignment, completed in 30 minutes. The memory consumption increased at the start of the program execution, and was stable during the whole filtering, reaching a maximum value of 4,850kB (as measured by the maximum resident set size, see Additional file 2). The output file contained 3,591 trees (one for each window). Among those trees, 613 grouped Human and Gorilla and 547 grouped Chimpanzee and Gorilla as the closest relatives leading to an estimate of 32% incomplete lineage sorting. This value is very similar to what was reported using more advanced modelling on the same data set [9].

### Extract homologous regions of a gene set

**Figure 4:**
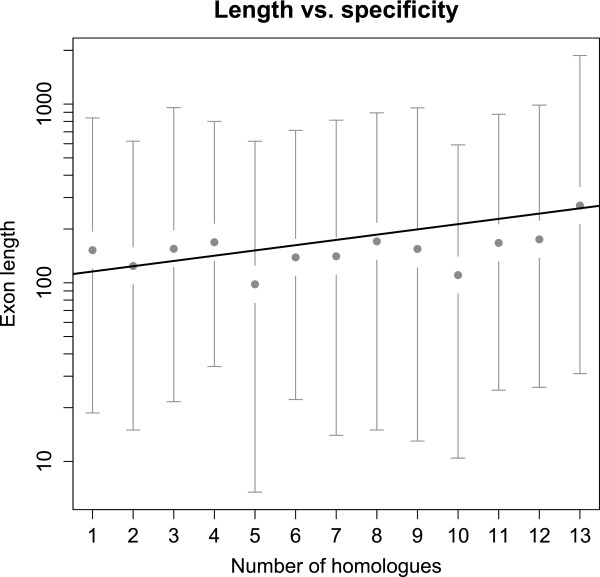
***Zymoseptoria tritici *exon length.** Exon length plotted as a function of the number of strains for which a homologous exon is found. Grey points and bars represent the observed means and the 2.5 and 97.5% quantiles of the observed distributions, respectively. The solid line is the estimated regression line.

We analysed the multiple alignment of the fungus *Zymoseptoria tritici* (synonym *Mycosphaerella graminicola*) aligned with 12 genomes of three closely related species [11]. We extracted all sequences homologous to the reference gene set from *Z. tritici*, containing 28,314 exons. MafFilter extracted all features where at least one homologue was found and output the resulting alignments in a file with Clustal format. General statistics such as the number of homologues found were also computed. The full analysis completed in less than five minutes and used 30MB of memory (See Additional file 2). The pipeline extracted 24,932 exons, of which 24,514 had at least one homologue in another genome, and 20,268 were shared by all the 13 aligned genomes. We report a highly significant positive correlation between the exon length and number of homologous exons (Figure 4, Kendall’s *τ* = 0.133, p-value < 2.2e-16) suggesting that isolate/species-specific genes tend to be shorter. Among short genes are the so-called effector genes which play a role in pathogenicity by manipulating the host defence and metabolism. This result is therefore consistent with a high dynamics and specificity of the effector gene repertoire.

### Extract all non-coding regions from a single genome

**Figure 5:**
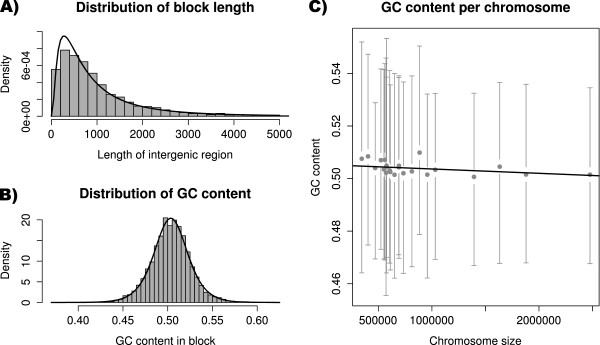
***Ustilago maydis *intergenic regions.A)** Distribution of the length of intergenic regions in the *U. maydis* genome (histogram) with the corresponding fit of a log-normal distribution (solid line). **B)** Distribution of GC content in intergenic regions (histogram) with the corresponding fit of a logistic distribution (solid line). **C)** GC content plotted as a function of chromosome size. Grey points and bars represent the observed means and the 2.5 and 97.5% quantiles of the observed distributions, respectively. The solid line is the estimated regression line.

In this example, a Fasta file containing all chromosome sequences of the fungus *Ustilago maydis*[12,13] was imported and compared to a GFF3 annotation file containing all protein coding genes [14]. Fasta import is enabled in MafFilter providing that the sequences are comprehensive, that is, all contig data start at nucleotide position 1. In this pipeline all gene sequences defined in the GFF files were removed. The resulting blocks were then filtered to keep only regions greater than 300 bp, and were written in a gzip-compressed MAF file. Statistics (block length, nucleotide composition) were also computed on the resulting blocks. The program completed in 18 minutes, used a maximum of 86MB of memory (See Additional file 2) and output 5,487 blocks of intergenic regions. The global distribution of intergenic region lengths was well fitted by a log-normal distribution with mean 6.542, corresponding to an intergenic length of 694bp (Figure 5). The GC content followed a logistic distribution with location 0.503 and scale 0.012, revealing a homogeneous GC content along the genome. GC content, however, was weakly – yet significantly – negatively correlated with chromosome size (Figure 5, Kendall’s *τ* = -0.034, p-value = 0.0002603). Such a correlation is expected if GC-biased gene conversion is at stake since smaller chromosomes have a higher average recombination rate and therefore a higher GC-content equilibrium [15].

## Conclusion

Integrated analysis of large genome alignments is a computational challenge for today’s comparative and evolutionary genomics research and its importance is expected to grow in the near future. We have introduced here the MafFilter program that allows the easy and efficient analysis of such data. The program is highly parametrizable and allows to perform a broad range of analyses and data processing on MAF files. In addition, the components of the underlying parsers and methods are available as an object-oriented library, facilitating the implementation and integration of new analysis tools. As it reads and outputs standard formats, the MafFilter software is a powerful complement of existing genomic tools such as the SAMTools [16] and VCFTools [17].

## Availability and requirements

### MAF parser and filters

**Project name:** MafFilter

**Project web site:**http://bioweb.me/maffilter

**Operating systems:** all for which a C++ compiler is available, including GNU/Linux, MacOS and Windows

**Programming language:** C++

**Compiler:** gcc 3.4 and higher versions

**Other requirements:** the C++ standard library, the bpp-core, bpp-seq, bpp-phyl, bpp-seq-omics and bpp-phyl-omics libraries from Bio++ (available at http://bioweb.me/biopp), the boost-iostreams library (available at http://www.boost.org/users/download/).

**License:** open source software distributed under the GPL-compatible CeCILL version 2.0 license.

The MAF parser and analysis filters (see Table 1) are available through the Bio++ libraries *bpp-seq-omics* and *bpp-phyl-omics*[6]. The *bpp-seq-omics* library contains the parser *sensu stricto* and the sequence based analysis tools, while the *bpp-phyl-omics* provides more advanced computational tools such as phylogenetic reconstruction methods. The documentation of the application programming interface (API) is available online on the Bio++ website at http://bioweb.me/biopp/articles/documentation/. The API is generic and enables the user to use the parser in his/her own software. It also allows the implementation and combination of new filters with the existing ones. A complete manual (PDF and HTML) is available from the MafFilter website, which describes all available options. Example application files are distributed along with the program.

## Competing interests

The authors declare that they have no competing interests.

## Authors’ contributions

JYD, SG and EHS designed the software. JYD and SG implemented the algorithms and developed the software. JYD and EHS wrote the manuscript. All authors read and approved the final manuscript.

## Supplementary Material

Additional file 1 BioPerl script using the SeqIO:Maf parser. Click here for file

Additional file 2 CPU and memory usage during the execution of MafFilter for the three example pipelines. Click here for file
