# Supplementary material for: MafFilter: a highly flexible and extensible multiple genome alignment files processor
Source: BMC Genomics. 2014 Jan 22;15:53. doi: 10.1186/1471-2164-15-53 (PMC3904536; doi:10.1186/1471-2164-15-53)

Example 1: Compute genetic distances

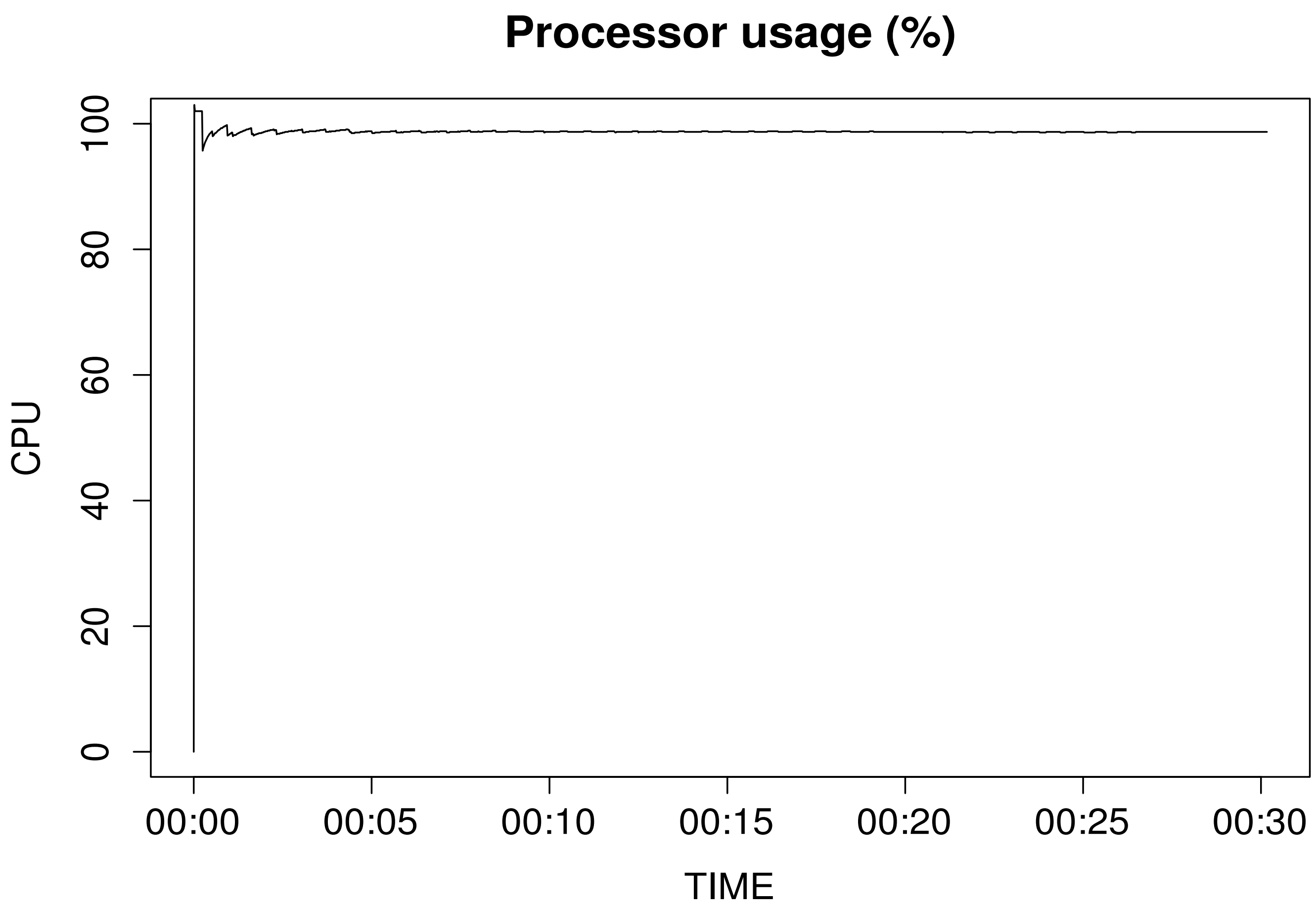

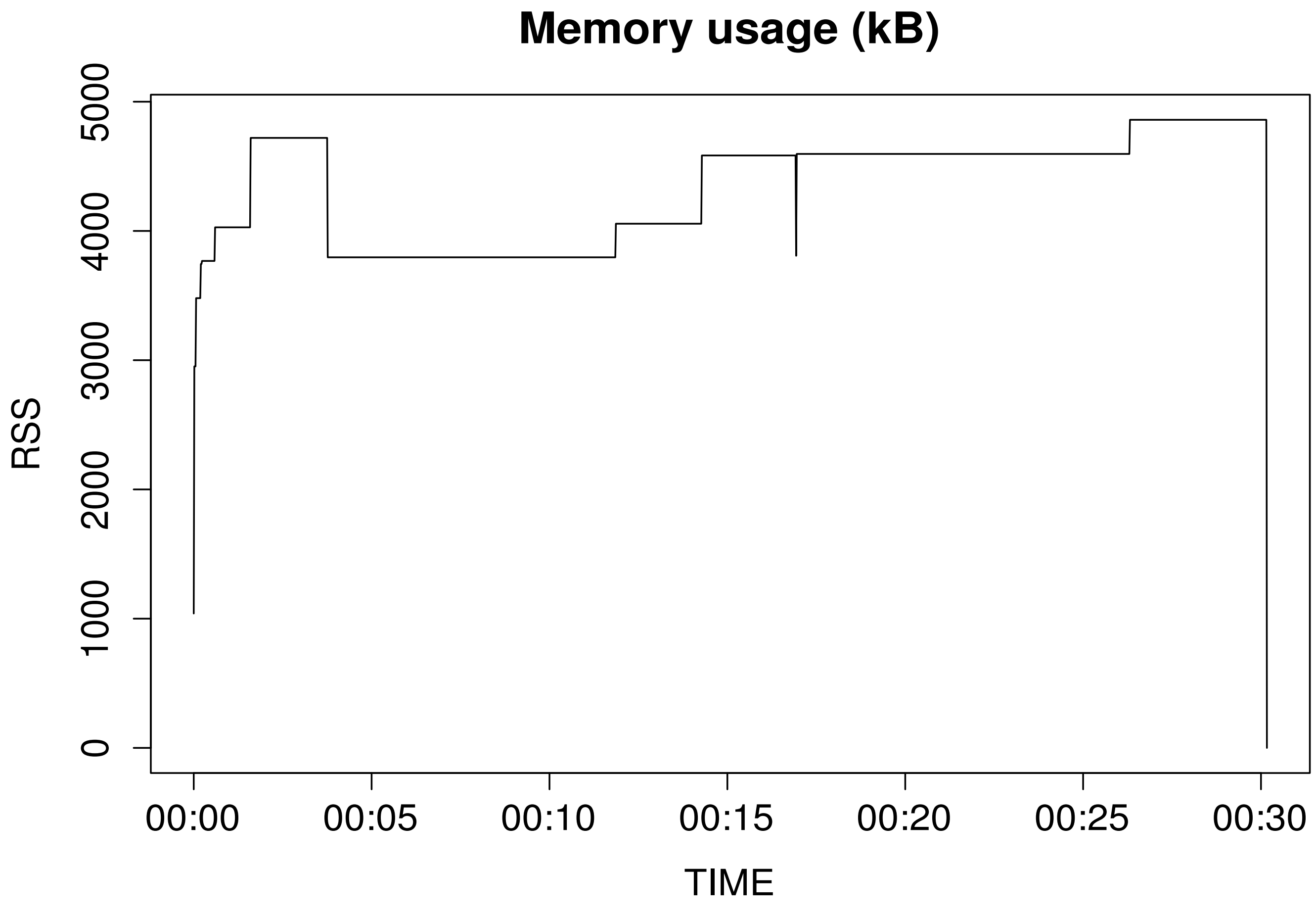

Example 2: Extract non-coding regions and compute GC content

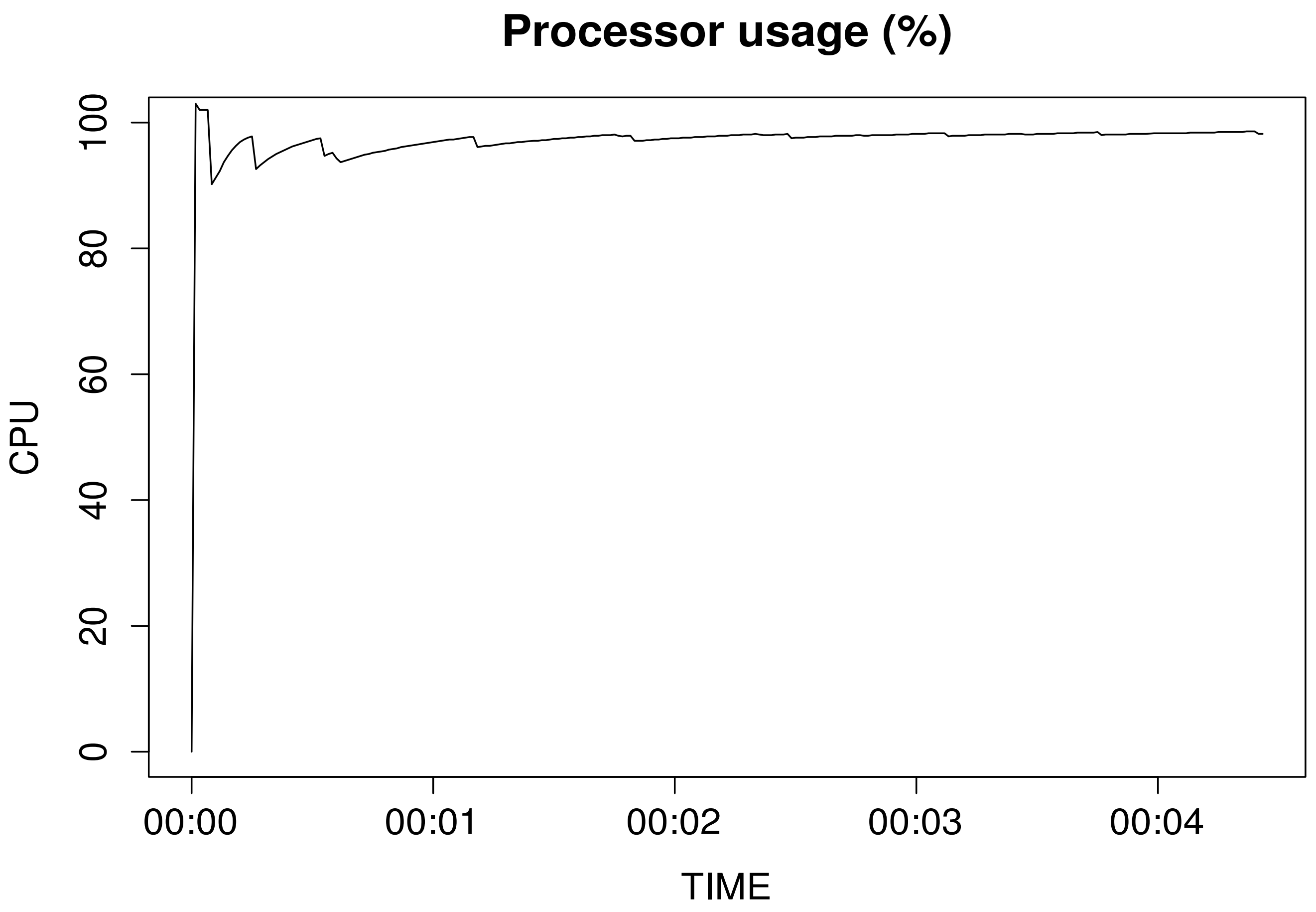

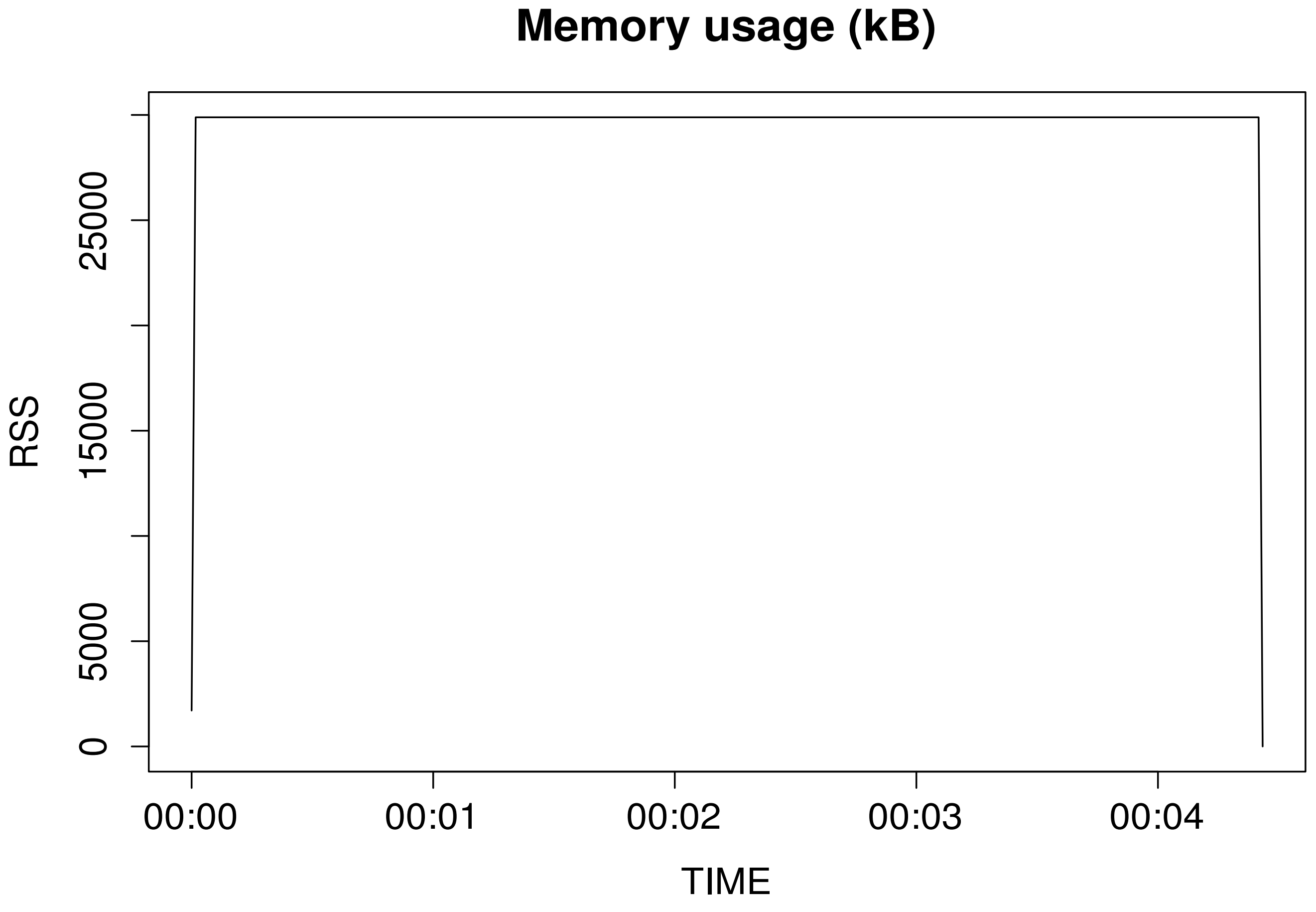

Example 3: Extract homologous gene sets

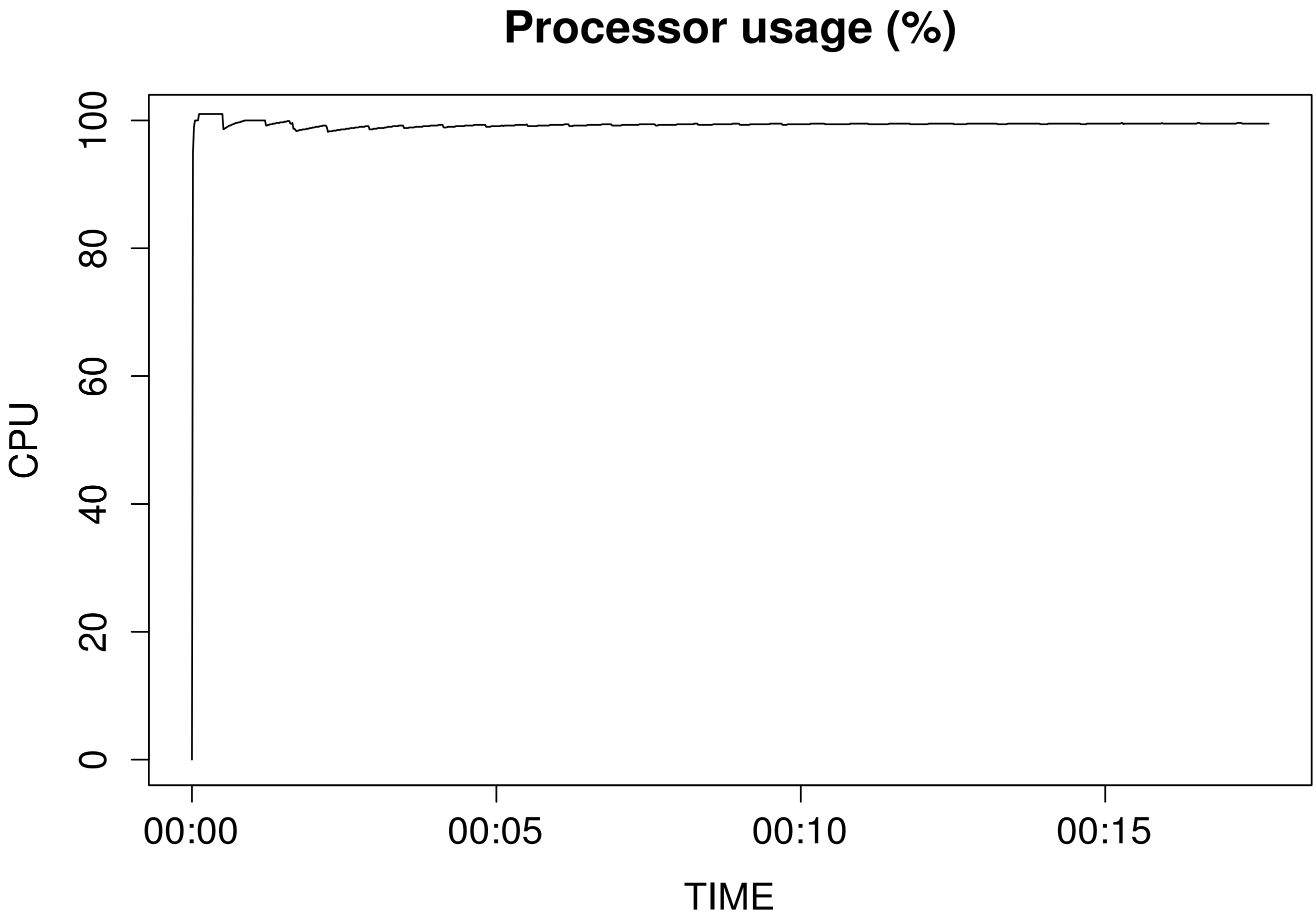

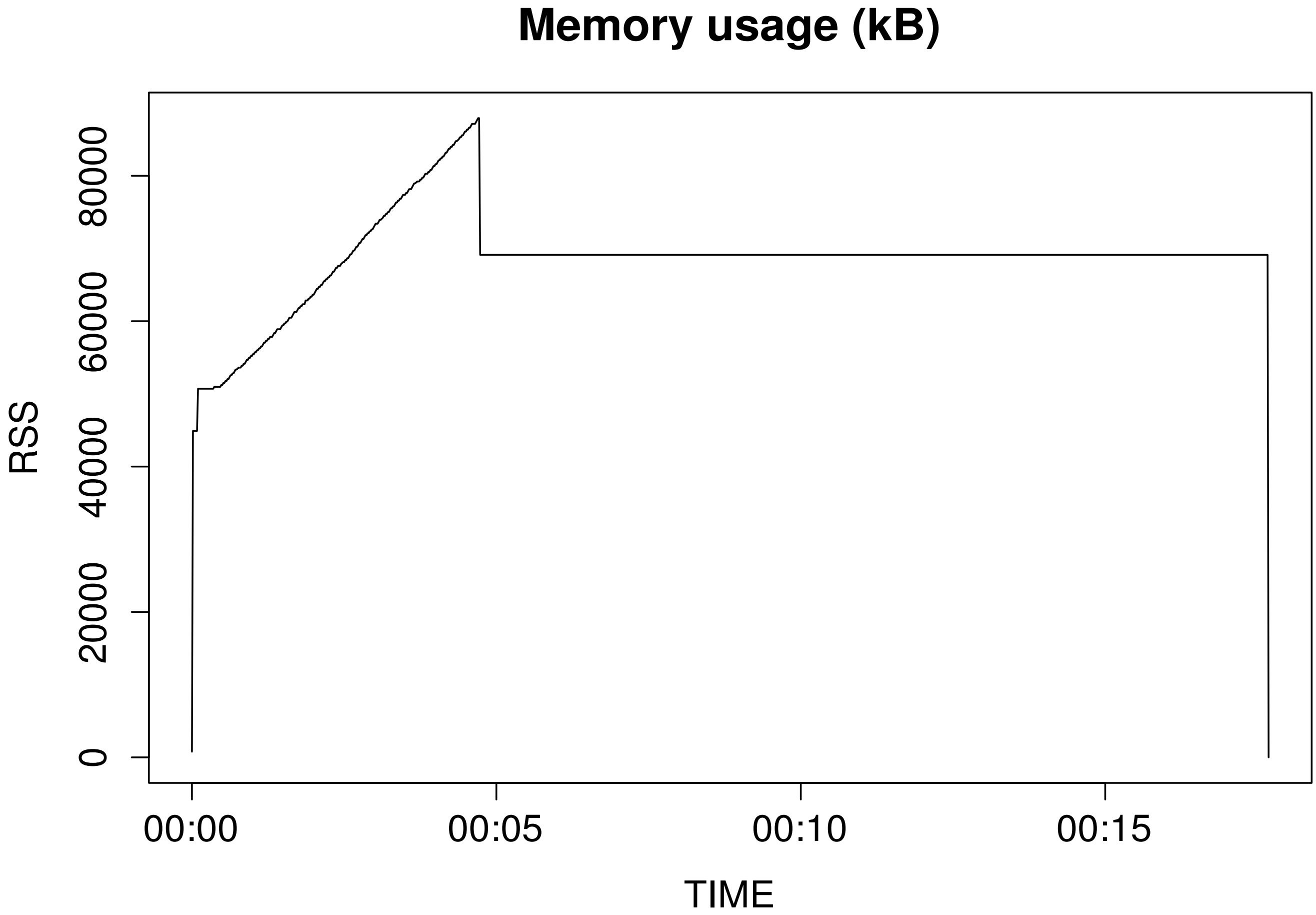

Supplement: Additional file 2 — CPU and memory usage during the execution of MafFilter for the three example pipelines. [file 1471-2164-15-53-S2.PDF]
